# Uranium and Associated Heavy Metals in *Ovis aries* in a Mining Impacted Area in Northwestern New Mexico

**DOI:** 10.3390/ijerph14080848

**Published:** 2017-07-28

**Authors:** Christine Samuel-Nakamura, Wendie A. Robbins, Felicia S. Hodge

**Affiliations:** 1 School of Nursing, University California, Los Angeles (UCLA), 4-246 Factor Bldg., Mailcode 691821, Los Angeles, CA 90095, USA; 2 Center for Occupational and Environmental Health Fielding School of Public Health, Environmental Health Sciences, UCLA, 5-254 Factor Bldg., Mailcode 956919, Los Angeles, CA 90095, USA; wrobbins@sonnet.ucla.edu; 3 School of Nursing, UCLA, 5-940 Factor Bldg., Mailcode 691921, Los Angeles, CA 90095, USA; fhodge@sonnet.ucla.edu

**Keywords:** sheep, contamination, food chain, mining, Navajo, heavy metals, uranium, Cadmium, Molybdenum, Selenium

## Abstract

The objective of this study was to determine uranium (U) and other heavy metal (HM) concentrations (As, Cd, Pb, Mo, and Se) in tissue samples collected from sheep (*Ovis aries*), the primary meat staple on the Navajo reservation in northwestern New Mexico. The study setting was a prime target of U mining, where more than 1100 unreclaimed abandoned U mines and structures remain. The forage and water sources for the sheep in this study were located within 3.2 km of abandoned U mines and structures. Tissue samples from sheep (*n* = 3), their local forage grasses (*n* = 24), soil (*n* = 24), and drinking water (*n* = 14) sources were collected. The samples were analyzed using Inductively Coupled Plasma-Mass Spectrometry. *Results:* In general, HMs concentrated more in the roots of forage compared to the above ground parts. The sheep forage samples fell below the National Research Council maximum tolerable concentration (5 mg/kg). The bioaccumulation factor ratio was >1 in several forage samples, ranging from 1.12 to 16.86 for Mo, Cd, and Se. The study findings showed that the concentrations of HMs were greatest in the liver and kidneys. Of the calculated human intake, Se Reference Dietary Intake and Mo Recommended Dietary Allowance were exceeded, but the tolerable upper limits for both were not exceeded. Food intake recommendations informed by research are needed for individuals especially those that may be more sensitive to HMs. Further study with larger sample sizes is needed to explore other impacted communities across the reservation.

## 1. Introduction

Ethnically diverse populations are disproportionately exposed to hazardous environmental materials by virtue of living in close proximity to toxic waste materials [1]. One-half of the uranium (U) in the US is found on American Indian (AI) lands, where mining, milling, processing, and waste storage has commonly occurred. From the 1940s to the 1980s, northwestern New Mexico (NM) alone contributed 40% of the U.S. U production [2]. The study setting was a prime target of U mining for military purposes from the 1940s to the 1980s.

Diné (Navajo) lands were one of the prime targets for mining, contributing thirteen million tons of U ore for military use from 1945 to 1988 [3] and leaving more than 1100 abandoned and partially unreclaimed U mines, mills, and waste piles [4]. The extent of the health threats to the Diné community exposed to these sites is anticipated to be high. Uranium enters the body primarily by inhalation or ingestion (contaminated water or food), and then it enters the bloodstream and is deposited in tissues, primarily the kidneys and bones [5]. Human and animal studies of those exposed to U have shown kidney toxicity [6,7], as well as damage to the liver, muscles, cardiovascular, and nervous systems [5,6,7,8]. Arsenic (As) is a teratogen [9]. Cadmium (Cd) can accumulate in organs and impair renal function [10]; Lead (Pb) is associated with adverse effects on the nervous, developmental, renal and reproductive systems [11]. Selenium (Se) toxicosis can cause neurological and gastrointestinal problems [12] and endocrine function disruption [13] and is a teratogen in several species of animals [14]; Molybdenum (Mo) has been shown to be a male reproductive toxicant in animals and humans [15,16,17]. 

The Dine Network for Environmental Health (DiNEH) study worked closely with 20 Navajo chapters or communities to address the concerns of the community and leaders regarding the health effects of environmental exposures to unreclaimed U mines and mill sites [18]. The DiNEH cohort found that 40% of participants lived within 3.2 km of an abandoned U mine, 16% lived near a U mill [18], and 12.6% of children played on tailing piles or waste dumps [4]. In self-reported data of past exposure, 15.4% utilized materials from abandoned U mine sites to build homes or other structures, 1.8% sheltered livestock in abandoned mines, 12.7% herded their livestock near contaminated sites, and 12.8% said their livestock came into contact with contaminated water [4]. In this population, surface and groundwater utilization is important for human and livestock consumption as well as agriculture. In affected areas, greater than half of the Diné people continue to drink from unregulated water sources [18]. DiNEH data indicate that more than 80% of Diné people haul water for all uses, including irrigation and livestock watering, despite having regulated water in the home [18].

### Study Purpose and Aims

Mutton or lamb meat and organs are primary food staples in this community [19], and all aspects of the animal are used; there are important cultural uses for the animal. The purpose of this study was to determine if sheep, a harvested primary dietary food staple (and its forage and water sources) on Diné lands in northwestern NM, were contaminated with U and other associated heavy metals. Past studies of these areas in NM demonstrated that the consumption of U contaminated food may occur through the ingestion of locally raised livestock [20,21,22] and by way of their forage [20,21]. Food chain contamination in locally harvested food in the Diné community in NM was reported as a plausible exposure pathway [18]. The current study was undertaken to reexamine and contribute more recent data and introduce data not previously reported (heavy metal concentrations in wool, intestine, lung, and various biota).

## 2. Materials and Methods

This was a descriptive, comparative study examining contamination levels in locally harvested *O. aries*, their forage, and associated soil and water from reservation areas within a 3.2 km radius of previously mined areas. Data obtained from the DiNEH study cohort [18] served as one of the sources for identifying subjects and samples of food, herbs, water, forage, and soil. Additional participants were recruited by word-of-mouth, home visits, and advertising at public tribal community events. Of the DiNEH cohort respondents, those individuals who reported harvesting sheep were recruited for potential participation in the present study. Sheep chosen represented a range of ages (0.66–3.25 years), their proximity to mining structures, and a variety of water sources. Three ewes were included in this study. The individual sheep data are compared and reported to reflect an accurate measure of heavy metal (HM) uptake in *O. aries* tissue with respect to the associated forage, water consumption, and their environment.

### 2.1. Study Setting

This study was reviewed and approved by the University of California, Los Angeles (UCLA), the Institutional Review Board (IRB# 11-001594-CR-00005), The UCLA Office of Animal Research Oversight (OARO, #2011-065-03), and the Navajo Nation Human Research Review Board (#NNR-11.321).

**Figure 1:**
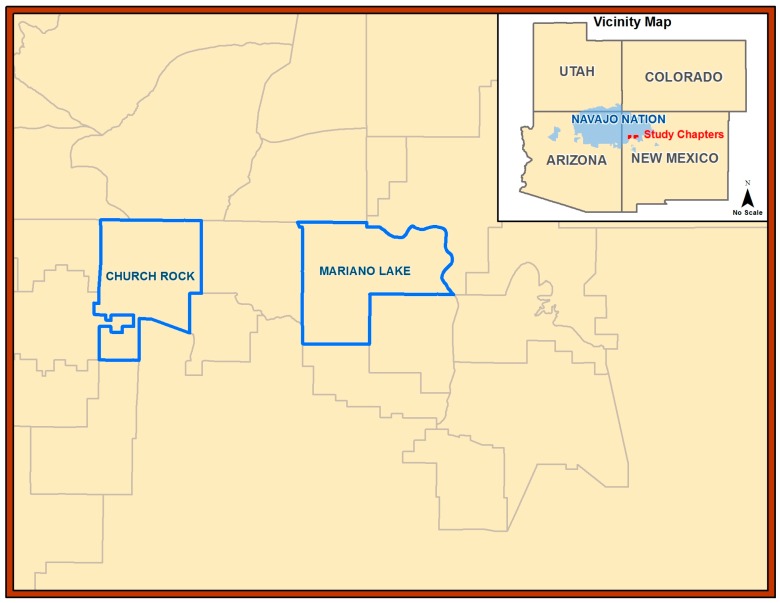
Study area map. Depiction of the Navajo Nation in the Four Corners region of the U.S. Southwest. New Mexico Chapters or communities; Churchrock and Mariano Lake provided sheep tissue and associated samples.

The study area is a semi-arid to arid region of the American Southwest in northwestern NM on Diné reservation lands (Figure 1). The average precipitation was less than 25 cm per year according to meteorological data for NM (Western Regional Climate Center Western U.S. Climatic Historic Summaries) for the study period. Despite several decades of longstanding drought in the area, community members still participated in subsistence activities. Two “Chapters” provided sheep and associated samples. The Mariano Lake Chapter is 272 km^2^ of land mass and the Churchrock Chapter is 233 km^2^ (total land mass of 505 km^2^). Recruitment was initiated on May 2012 and enrollment began in July 2012. All samples were collected from 10 November to 13 December 2012. This study focused on sheep as a food staple and was part of a larger “parent” research project that examined subsistence farming on the reservation, including the metal contamination of herbs [23].

### 2.2. O. aries Tissue Samples

The sheep tissue samples were collected when a sheep rancher harvesting session took place. Three sheep came from two different chapters. From 10 November to 13 December 2012, three ewes (eight months, three, and 3.25 years of age) were contributed to the study. The *O. aries* tissue samples were collected in the field immediately after slaughter and included muscle, bone, intestine, lung, liver, kidney, and wool. Upon collection, all samples were placed on dry ice and shipped to the University of New Mexico (UNM) Analytical Chemistry Laboratory Earth and Planetary Sciences Department for storage and analysis. The 13th cortical rib bone samples were sheared from the proximal, middle, and distal portions and composited together after the removal of excess tissue. The proximal, medial, and distal portions of the small intestine were collected and composited. For lung tissue, the samples were derived from each anatomical lobe and composited. Both kidneys were sampled, and the cortex and medulla were composited separately. Composited muscle samples were from the proximal, medial, and distal portions of the gastrocnemius. Of the wool fiber samples, the area over the neck, middle section, and posterior portions of the animal were sampled and composited. All tissues were representative of 1 g of dried tissue. For coupled organs, the tissues collected from the right side of the sheep were labeled as the sample, and one duplicate was obtained for each tissue type from the left side of the animal. A composited duplicate or replicate was obtained for non-dual type organs (liver and intestine).

### 2.3. Soil Samples

A stainless steel hand auger with a Teflon^®^ coated-core sampler was used to collect the soil samples. To minimize cross contamination, a polyethylene (PE) core liner was utilized. Soil samples were obtained from the topsoil (0–15 cm) and composited. The forage soil samples were obtained by utilizing a topographic soil zone sampling pattern using a random zig-zag pattern. Soil samples were weighed at 100 g. Physicochemical properties such as temperature, pH, Munsell color, depth, and moisture were obtained. The sheep tissue samples were paired with the forage, soil, and water samples.

### 2.4. Biota Samples

Live plants (edible forage portion, and roots) were removed from the ground soil and handpicked with a latex gloved hand, stored in PE plastic bags, and immediately placed on dry ice. The plant sources were non-cultivated. Each plant was divided into above ground and root portions. The plant roots were gently washed with deionized water (American Society Testing and Materials II heavy metal grade). The samples were weighed, photographed, bagged, and placed on dry ice for shipment. Due to the collection of sheep tissue and forage late in the harvesting season, non-nascent forage comprised most of the samples. The live plants were placed in a plant press for several weeks with daily press tightening. The dried samples were sent to the UNM Herbarium for identification and archiving.

For those plant forage and soil samples collected, the metal bio-accumulation factors (BF) were calculated as:

[BF = metal concentration in plant (*mc_plant_*)/metal concentrations in soils (*mc_soil_*)] (1)

The bio-accumulation factors are expressed on a dry weight (DW) basis.

### 2.5. Water Samples

The water samples were collected as a composite grab sample, except for those samples directly collected from a faucet or spigot were collected as first-draw samples. Lab grade PE water bottles were used, the volume of each sample collected was 250 mL. Chemical and physical characteristics data were collected (pH and temperature). Nitric acid (HNO_3_) preservative was added to each water sample, and the sample was immediately placed on dry ice. A duplicate for each sample was obtained. A blank for each sampling session was collected.

### 2.6. Statistical Analysis

International Business Machines (IBM) Statistical Package for Social Sciences (SPSS) for Windows, V. 21 (IBM Corp., Armonk, NY, USA) was utilized for statistical analysis. The concentrations of HMs found in sheep tissue, plant, and soil samples are reported in milligrams (mg) per kilogram (kg), DW. Heavy metal concentration levels are reported in micrograms (μg) per liter (L) for water samples. Percentages, range, mean (M), standard deviation (SD), and median were used to summarize the data. Independent *t* tests compared HMs in root versus above ground plant, root soil, and topsoil. Correlations and linear regression tested for associations between sheep tissue and associated water and forage.

### 2.7. Sample Preparation and Analysis

The field samples arrived on dry ice and were stored in a −20 °C freezer until sample preparation and analysis. The organic samples were oven dried at 65 °C. Upon sampling the debris were removed from the wool fiber. The wool was washed with 18 mega Ohm water then soaked in dilute (0.01 N) HCl. The wool was dried in the oven at 65 °C. The samples were prepared by weighing two g dry mass into the digestion tube. Two mL Hydrogen peroxide (H_2_O_2_) and five mL HNO_3_ were added, and the samples were heated gradually up to 95 °C and digested for two hours. Next, the digested samples were transferred into 50 mL volume metric flasks and brought to volume using 18 mega ohm water. Three mL of HNO_3_ (reagent blank) was run with each batch of samples.

The samples were analyzed using PerkinElmer NexION 300D ICP/MS by diluting 100 times (100× D.F.) in glass culture tubes. Mixed standards (As, Cd, Mo, Pb, Se, and U) were prepared using single element standards. The calibration standards range was 5, 10, 25, and 50 μg/L (ppb). The Quality Control (QC) samples were comprised of Initial Calibration Blank Verification (ICBV), Initial Calibration Verification (ICV), Continuing Calibration Verification (CCV), and Matrix Spike (MS), Matrix Spike Duplicate (MSD), and Matrix Spike Replicate (MSR). The method detection limits are as follows: As 0.3 μg/L, Cd 0.1 μg/L, Mo 0.02 μg/L, Pb 0.008 μg/L, Se 1.3 μg/L, and U 0.008 μg/L.

A mixed internal standard of Bismuth, Indium, Scandium, and Yttrium (Bi, In, Sc, Y) was used to match the analyte mass range. Two percent HNO_3_ was used as a carrier and rinse solution. The elements were analyzed in three modes to minimize interferences; Standard, Dynamic Reaction Cell gas A (Anhydrous Ammonia), and Dynamic Reaction Cell gas B (Oxygen) in groups. Upon the completion of analysis, the data were revised, validated, and tabulated, and the concentrations were converted to mg/kg material using instrument corrected concentration readings, sample digest final volume, and sample weight.

For each sample, three replicates were measured. The accuracy of the method was verified using the analysis of certified reference materials National Institute of Standards and Technology (NIST) Standard Reference Material (SRM) 2709a San Joaqin Soil (NIST, Gaithersburg, MD, USA) and NIST SRM 1573a (Tomato Leaves) (NIST, Gaithersburg, MD, USA), yielding the following values: Cd: 1.474 ± 0.107 mg/kg (reference tomato leaves value: 1.52 ± 0.04 mg/kg) and Cd: 0.644 ± 0.089 mg/kg (reference soil value: 0.371 ± 0.02 mg/kg). The precision results were satisfactory with relative standard deviations ranging from 7.3 to 13.8%.

## 3. Results

### 3.1. Human Harvester Questionnaire

The mean age of adult sheep harvesters was 58.67 ± 2.89 years; two of three participants were male. All sheep parts (liver, kidney, intestine, soup bone, lung, and muscle meat) were consumed by the participants for a mean of 52.33 ± 10.78 years. The sheep harvesters reported that 35% of their overall meat intake came from sheep they raised and harvested locally. On average, all the participants reported consuming locally raised sheep once a week.

The local harvesters reported other important non-food uses for sheep. All participants reported selling wool, and two reported using the locally harvested wool to create textiles to sell for income. One of three harvesters reported selling live sheep to market, and two reported selling sheep or lamb cuts to market. All the participants reported sharing sheep meat for free with others. On average, each sheep harvester distributed free meat to two households. Multiple sheep parts were reportedly used for various ceremonial or cultural purposes by all harvesters.

### 3.2. Sheep 1

Sheep 1 was an eight month old lamb and the youngest of the three sheep sampled in this study. This lamb grazed within a 3.2 km radius of three former U mines and a decommissioned U milling building. The grazing area was downstream of a major U tailings pond spill that occurred in 1979. For this ewe lamb, 75% of water consumption was from a public water source and 25% from an earthen dam. Among the three sheep in this study, the U concentrations in water were highest for this sheep. However, the difference between sheep did not reach statistical significance, *p* > 0.05. The recommended standards for the upper limits of potentially toxic contaminants in sheep drinking water were not exceeded for any of the HMs studied for this lamb (Table 1). The HM concentrations in water (*n* = 4) for Sheep 1 were U (8.24 ± 2.41 μg/L), Pb (7.60 ± 2.51 μg/L), Se (4.78 ± 1.25 μg/L), Mo (2.87 ± 0.78 μg/L), As (0.77 ± 0.32 μg/L), and Cd (0.26 ± 0.07 μg/L). Sheep 1 primarily foraged on three species of grass: *Achnatherum hymenoides* (Roemer and Schultes) Barkworth, *Bouteloua gracilis* (Wildenow ex Kunth) Lagasca ex Griffiths, and *Pascopyrum smiithii* (Rydberg) Löve. While some differences in HM concentrations were found across the forage species, no HMs exceeded the maximum tolerable concentrations set for sheep intake (Table 2) for above the ground portions and root portions (Table 3).

For Sheep 1, the forage BF ratio for all metals ranged from 0.08 to 15.0. The Cd ratio was 1.6 in *A. hymenoides* and 1.17 in *B. gracilis*. The BFs for Mo was 15.0, 7.9, and 2.70 for *A. hymenoides*, *B. gracilis*, and *P. smithii*, respectively.

In tissues of this sheep, Se concentration in the liver was 3.93 mg/kg (Table 3), in the kidney medulla 2.04 mg/kg, the cortex 0.53 mg/kg, the wool 1.30 mg/kg, and for the remainder of organs, the concentrations ranged from between 0.39 mg/kg (lung) to 0.77 mg/kg (intestine). The Pb concentrations for wool and bone were 1.12 and 0.20 mg/kg. For Mo, the liver concentration (1.20 mg/kg) was greater than the kidney medulla (0.43 mg/kg) or cortex (0.05 mg/kg), *p* > 0.05. The As, Cd, and U tissue concentrations were lower (bone: 0.20 mg/kg, wool: 0.06–0.66, intestine: 0.01 mg/kg, liver: 0.06–0.001 mg/kg, medulla: negligible–0.09 mg/kg, and cortex: negligible–0.08 mg/kg), *p* > 0.05.

### 3.3. Sheep 2

Sheep 2 was three years old. This sheep grazed within a 3.2 km proximity of three former U mines and within the same downstream area of a historical U tailings pond spill area as Sheep 1. Of the water sources for this sheep, no HM concentrations exceeded the recommended standards for the upper limits of toxic contaminants set for livestock drinking water (Table 1). The HM concentrations in water (*n* = 4) for Sheep 2 were Pb (7.98 ± 1.47 μg/L), Se (6.23 ± 0.41 μg/L), U (4.93 ± 0.23 μg/L), Mo (1.94 ± 0.07 μg/L), As (1.01 ± 0.30 μg/L), and Cd (0.65 ± 0.51 μg/L). Sheep 2 drank primarily from a public water source (75%) and from an earthen dam (25%). Sheep 2 foraged primarily on four species of plants: *Aristida purpurea* Nuttall, *B. gracilis*, *Muhlenbergia repens* (Presl) Hitchcok, and *Pleuraphis jamesii* Torrey. The averages for heavy metal concentrations for the above ground portions of forage did not exceed the maximum tolerable concentrations (Table 2). Across all metals, the BF ranged from 0.09 to 14.5 for Sheep 2 forage and was greater than 1.0 for Cd in *A. purpurea* (2.67), *B. gracilis* (1.13), and *P. jamesii* (1.88). Greater ratios were seen in Mo for *A. purpurea* (14.5), *B. gracilis* (8.33), *P. jamesii* (2.94), and *M. repens* (4.3).

In general, of the three sheep, Sheep 2 had the lowest concentrations of organ HMs, but these differences did not reach statistical significance. However, this sheep had the second highest concentration of Se in both the liver (5.93 mg/kg) and kidney medulla (2.83 mg/kg; Table 4). Intermediate concentrations were found for As, Cd, Pb, and Mo (bone 1.09 mg/kg, lung 0.25 mg/kg, liver 0.22–1.39 mg/kg, muscle 0.10 mg/kg, wool 0.04–1.07 mg/kg, medulla 0.10–1.02 mg/kg, and cortex negligible–0.25 mg/kg), *p* > 0.05. The lowest organ concentrations were found in U (0.08 mg/kg in wool, 0.001 mg/kg in medulla, 0.01 mg/kg in lung, and negligible levels in the renal cortex), *p* > 0.05.

### 3.4. Sheep 3

Sheep 3 was the oldest sheep, sampled at 3.25 years of age. This sheep grazed within a 3.2 km radius of two mines. Sheep 3 drank exclusively from unregulated waters sources such as a livestock water source 60% of the time and 20% each from an earthen dam and private well. The concentration of As in the drinking water of Sheep 3 was significantly higher than that of either Sheep 1 or 2 (*p* < 0.001). However, no HM concentrations exceeded the recommended standard for the upper limits of toxic contaminants for livestock drinking water (Table 1). The HM concentrations in water (*n* = 6) were Pb (7.49 ± 2.30 μg/L), Se (6.29 ± 2.75 μg/L), Mo (4.42 ± 2.35 μg/L), U (3.77 ± 3.43 μg/L), As (1.25 ± 0.91 μg/L), and Cd (0.03 ± 0.20 μg/L). The primary forage materials for Sheep 3 were *B. gracilis* and *Sporobolus cryptandrus* (Torrey) A. Gray, and the heavy metal maximum tolerable concentrations were not exceeded.

The Sheep 3 data showed that the BF ratios for HMs ranged from 0.15 to 16.86. The cadmium for both *B. gracilis* (1.29) and *S. cryptandrus* (2.13) was >1.0. For Mo, the ratio was 16.86 for *B. gracilis* and 1.10 for *S. cryptandrus*. The Se BF was found to be 1.14 in *B. gracilis*.

For Sheep 3, the Se level was 3.28 mg/kg in the liver and 2.62 mg/kg in the kidney medulla (*p* > 0.05; Table 5). In the wool, the highest concentration was found in Pb (1.90 mg/kg), *p* > 0.05. Middle range concentration levels were found with As, Pb, and Mo (bone 0.70 mg/kg, wool 0.23–1.90 mg/kg, liver 0.14–1.47 mg/kg, medulla 0.09–0.47 mg/kg, and cortex 0.07–0.11 mg/kg), *p* > 0.05. The uranium concentrations were lowest in the sheep’s organs (wool 0.09 mg/kg, lung 0.01 mg/kg, kidney medulla 0.002 mg/kg, and renal cortex 0.001 mg/kg), *p* > 0.05.

### 3.5. Sheep Forage

Seven species of forage were collected and identified. These were non-cultivated forage, and the participating harvesters did not report the use of fertilizer or any other type of natural or commercial soil amendments. Depending on the number of forage species collected in this study, the calculated sheep average intake ranged from 5.97 to 34.38 g of dry matter (DM) for Sheep 1, 4.48–25.24 g of DM for Sheep 2, and 11.23–49.44 g of DM for Sheep 3. There was some forage consumption overlap amongst the sheep grazing areas. The most abundant forage in the study areas was *B. gracilis* or blue grama. Of all the forage samples, *B. gracilis* comprised the most numerous samples at 46%, 15% was attributed to *P. smithii* (western wheatgrass), and 8% was split equally between five local forages (*A. hymenoides* or Indian ricegrass, *A. purpurea* or purple threeawn, *M. replens* or creeping muhly, *P. jamesii* or galetta, and *S. cryptandrus* or sand dropseed). All sheep forage was collected within a 3.2 km proximity of mines and features. The various forage species demonstrated different propensities for certain heavy metals. For instance, *A. purpurea* contained higher levels of Pb (2.66 mg/kg) and Se (2.31 mg/kg), while *M. repens* showed greater uptake of Pb (1.96 mg/kg) and Se (1.33 mg/kg). *Pleuraphis jamesii* showed higher levels of Se (2.41 mg/kg) and Pb (2.15 mg/kg). Also, Se (2.28 mg/kg) and Pb (2.00 mg/kg) showed the greatest uptake in sand dropseed. Compared to the other heavy metals in forage, U consistently had relatively low concentrations except for in *A. hymenoides* (0.43 mg/kg) and *P. smithii* (0.57 mg/kg). The great distribution of *B. gracilis* allowed a comparison in all three sheep forage areas. In most instances the root of each plant contained greater concentrations of HMs than the above ground portion of the forage. For example, five of seven plants showed that the Mo concentrations in the above ground portions of forage were higher than in the root portions.

## 4. Discussion

The existing literature reports HM levels in kidney tissue, but typically there is no comparison between the kidney medulla and the renal cortex. In this study, the kidney medulla rather than the kidney cortex showed an increased uptake of U, Se, Mo, and As. The renal proximal tubule epithelia are chemically damaged by high acute levels or prolonged low doses of U [24,25]; the proximal tubules are housed in the renal cortex. The administration of toxic doses of Se demonstrated histopathological changes in the proximal tubules of the sheep [26]. The kidneys maintain Se homeostasis [27]. Renal compromise may cause dysregulation of Se. Our study indicates there may be a difference between HM accumulation in the medulla and the cortex. The renal toxic effects of U and Cd are well supported in the literature [12,24]. The effects of associated heavy metals on the sheep kidney need further exploration.

Meat protein is richer in Se than plants [28]. The literature supports that Se commonly concentrates in the liver and kidneys of animals [29,30]. Of all sheep organs, elevated Se levels were found in the liver (3.28–5.93 mg/kg) and kidney medulla (2.04–2.83 mg/kg). In a lamb tissue study [31], it was reported that Se concentrations in the kidneys were seven to 44 times higher than in other tissue and organs. Similarly, in our study, the medullary levels contained the higher concentrations of Se (2.6–5.8 times higher than intestine and bone). The above lamb study reported that leg muscle contained the lowest Se concentrations of the tissues sampled [31]. We also found that leg muscle contained the lowest Se concentrations in our examination (0.45–0.76 mg/kg). There is a narrow margin between Se requirement and toxicity. Therefore, taking an accurate measure of food intake containing Se, particularly meat protein, is important. Food processing such as cooking via baking, boiling, and grilling may alter the amount of Se in food [13]. Whether food processing has an additive or minimizing effect on Se concentrations (and other HMs) in food is to be determined by research. Adjusting food intake and cooking habits based on various HM measurements and bioavailability may be a plausible intervention once it is informed by research.

Elevated levels of Se and Pb were found in sheep wool in the current study. Further, though Th was negligible in all other sheep tissue, it was detected in sheep wool (0.29 ± 0.70 mg/kg, 0.23–0.37 mg/kg; data not shown). This finding may indicate that Th (as well as other heavy metals) may be accumulating across time in sheep wool. Direct dirt and dust aerosol capture and the effects of lanolin may be contributing exposure factors. We did not measure the effects of lanolin in this study. The current study community relies on wool to create textiles. It is common practice to place local plants in hot water (drawn locally) to pigment the wool. The wool is handled often by weavers once the wool is removed from the animal, hand-carding the wool, hand-spinning, dyeing, and weaving the textile. The entire process often takes weeks to months, suggesting a potential lengthy human exposure to heavy metals. A considerable amount of time is spent outdoors for such activities, and exposure to various sources of contaminants such as soil, water, and air is a concern. Although this study of three sheep provided interesting insight, future studies should focus on determining the speciation of heavy metals and evaluate which metals have a greater affinity to wool.

The majority of the time for this study, heavy metals were found in the greatest amounts in soil > forage roots > above-ground forage parts, respectively. The current study mean Se soil concentrations were equivalent or exceeded the exposed soil and were greater than the control concentrations reported by Dreesen and Cokal (control: 0.7 mg/kg; tailings soil: 2.3 mg/kg) [32]. The above-ground forage parts contained the least amount of heavy metals, except for Mo and Cd. The bio-accumulation ratio (>1.0) can partially demonstrate the ability of particular plants to absorb soil heavy metals and transport them to the above ground portions of a plant. The data shows that the uptake of Cd, Mo, and Se by most plants sampled were high under current soil conditions. The highest BF ratios were seen in most forage for Mo (4.33–16.86), Cd (1.13–2.67), and Se (1.12–1.14), which needs further exploration. The high BF ratios seen may indicate a low tolerance of various plants (*B. gracilis*, *A. purpurea*, *A. hymenoides*, and *P. jamesii*) to high concentrations of Mo and Cd. In particular, *B. gracilis* the most abundant plant, was associated with elevated BF ratios for Cd, Mo, and Se.

Generally, in the biota samples there were greater heavy metal concentrations in the plant roots than the above-ground portions, which is consistent with several other plant studies that found that U translocates in greater amounts to the roots than the shoots [23,33,34,35,36]. Similar to the current study, Soudek et al. reported that U was more localized in the root system [33,34]. Uranium accumulation was less in grasses than root crops and *Brassica* spp. [36]. Uranium uptake was found to be 3.9 or 4.5 higher in the presence of phosphate deficiency [33]. The micro and macronutrients available in soil affected the uptake of Cd in one source [12]. Geochemical characteristics have an important influence on HM plant uptake. Future investigations can focus on the interactions between trace elements or other factors (soil characteristics, climatic conditions, etc.) that may demonstrate an influence on HM plant uptake.

Forage and water intake are important considerations in livestock, and soil ingestion must also be considered. In the current examination, the soil samples showed greater HM concentration than sheep tissue and forage samples. It has been demonstrated that sheep, a ground feeding animal, eat one to two percent of soil when good forage is available and about 18% when low quality forage is available [37,38]. It has been shown that sheep intake and digestibility of more mature plant material decreases with advancing maturity [39] due to greater effort and time in chewing by the animal; sheep selectively graze in high-quality forage areas when they are available [40]. The forage environment of the sheep in the current study area exhibited high stocking rates, sparse vegetation, and mature forage samples, which may have contributed to higher HM concentrations in forage. Dung analysis can evaluate the amount of inorganic material in sheep diets and may be useful in future studies in the current study area. 

Previous studies have reported the concentrations of HMs in sheep tissues, plants, and soil in the target study area (Table 6). In most categories, our study results were comparable to or less than what was previously found. The current study tissue measurements were below the exposure and control concentrations reported by Millard et al. and Ruttenber et al. in the 1980s [21,22]. No excess cancer risk was calculated to be attributed to eating sheep meat, liver, kidney, and soup bone by humans; researchers recommended continued monitoring at that time [21,22].

The highest HM metal concentration in the diet was used for each animal to calculate and compare to the maximum tolerable concentrations, and the lowest concentration for each HM was used to compare to the requirements for Mo and Se. The calculations are based only on the forage samples collected and are not representative of the complete sheep intake. Maximum tolerable concentrations are established for sheep intake for As Cd, Pb, Mo, and Se (Table 2) [30,42,43]. All study animals did not exceed the calculated maximum tolerable concentrations for As, Cd, Mo, and Pb. All study sheep met the Mo and Se dietary requirements. Liver is the organ of choice to diagnose Se deficiency [30], and concentrations less than 0.21 mg/kg in sheep liver are considered deficient [44]. All study sheep liver concentrations did not indicate deficiency. In sheep, Se toxicity was reported at 0.25 mg/kg of body weight (BW) chronically [30]. However, the National Research Council set the maximum tolerable concentrations for Se at 5.0 mg/kg of DM [43].

Of the donated sheep, the shepherds did not report indicators of acute (death, dyspnea, tachycardia) or chronic Se poisoning (alopecia, respiratory failure, hoof malformations, or lameness). Other supplementary sources of forage were not reported at the time of sampling; sheep harvesters reported relying on alternative fodder sources for their sheep in the late winter months only (outside the sampling window). New Mexico is one state that was reported to have high Se concentrations in soils [42] and those in areas with low annual rainfall or alkaline soil [30]. The mean study soil pH was 7.31 ± 0.51. Primarily, most of the Se is absorbed in the small intestines of ruminants and less absorption is seen in forage based diets versus diets based on concentrate [45]. Plants that accumulate Se (*Stanley pinnata*, *Haplopappus* spp., *Xylorrhiza glabbriuscula*, and *Astralgus* spp.) [46] may be unpalatable to grazing animals, but if there is lack of more palatable forage, animals may develop signs of toxicity from ingestion [30]. According to one study calculation, selenosis can occur in lambs ingesting 0.2% BW of Se accumulating plants [47]. Soil ingestion during foraging, seasonal soil forage adhesion (greatest in autumn and winter), pulling up of roots while foraging, and licking snouts by livestock may also contribute to higher HM concentrations [48]. The forage plants that we sampled were not known Se obligate or secondary accumulator plants [46]. Aside from Se, whether study plants accumulate Mo and Cd needs further evaluation.

Selenosis diagnosis is primarily based on Se measurements (in blood and urine), anemia and the presence of physical examination findings identifying toxic levels [30]. Selenium concentrations in the liver (5.93 mg/kg) and kidneys (2.83 mg/kg) were not elevated on a DM basis. One source reported that plant forage containing >3–5 mg/kg induced toxicity in sheep [49]. In our study, several plant roots exceeded 3 mg/kg, which is a concern with the pulling up of roots when sheep forage. The amount of root consumption in relation to the total sheep forage intake is important to determine. Further work examining these factors is an area of future research.

Based on drinking water standards for livestock, none of the heavy metal concentrations were above maximum tolerable concentrations (Table 1) [30]. Heavy metal water measurements collected by the DiNEH study (2007–2008) [50] from two of the water sources identified for Sheep 3 contained lower concentrations of Pb in comparison to our data (6.38–7.98 μg/L); the remaining HM data (As, Mo, Se, and U) were less than what the DiNEH researchers found. Most of the shepherds obtained public water for sheep consumption, which was reflected in the concentration levels found in sheep water. Harvesters in the study reported a history of consuming unregulated water intended for livestock. However, the As, Cd, Pb, Se, and U concentrations did not exceed the maximum contaminant levels set for human consumption (Table 1) [51,52]. The implementation of water use maps [18] may have contributed to the use of safer alternative water sources for these shepherds. Continued emphasis on the use of safe alternatives for water use in sheep and human consumption put forth by deLemos et al. [18] is essential.

Harvested food selling and sharing was common among the participants in the study. Emphasis should be placed on determining the incidence and frequency of food selling and sharing when assessing food chain contamination. Harvesting locations and activities can overlap in mining impacted areas. A few important factors to consider include the availability of harvest items based on seasonal variation and peak consumption periods (fall and winter season, important cultural activities, etc.). It is important to consider the consumption of contaminated food not only by individuals and their families but potentially the whole community and beyond [53].

### 4.1. Implications for Human Intake

Studies identified mutton as a core food staple [19,54], comprising 6% of the total energy and 10% of the total protein consumed in the Diné diet [54]. The current study participants reported that 35% of their total protein meat intake is comprised by local *O. aries.* The mean intake of local sheep protein was reported to be one day per week. In the study community, the typical serving size per foodstuff is reported to be 76.54 g of sheep muscle protein [55], roasted or boiled whole liver 377.5 g, roasted one whole kidney 76 g, and roasted or boiled lung 111.5 g [19]. Using the typical serving size for each food stuff, we used the maximum HM concentration for each food item to calculate the weekly intake of HM from the current diet of the study population. The Provisional Tolerable Weekly Intake (PTWI) limits are 15 μg/kg BW for As, 7 μg/kg BW for Cd, and 25 μg/kg BW for Pb [56,57], the study calculations were below the PTWI for As (1.64 μg/kg, 10.9% of PTWI), Cd (2.98 μg/kg, 42.5% of PTWI), and Pb (6.2 μg/kg, 24.8% of PTWI; Table 7). The intake of sheep protein containing Cd contributed 43% of the PTWI; conceivably, if an individual consumes locally harvested sheep protein more than 2.33 times a week, they will exceed the Cd PTWI.

The calculations for Mo and Se are based on the information provided by harvesters and are reflective of the sheep meat average consumption of one day per week. The Recommended Dietary Allowance (RDA) for Mo (45 μg/day) [58] was exceeded by more than a factor of 2 but, but the tolerable upper intake limit (UL) was not exceeded. The liver alone exceeded the Mo RDA by a factor of 1.8. By our estimates, an individual would have to consume half of the typical serving size to meet the RDA. The Mo levels in all sheep food products consumed comprised of 4.6% of the UL. The Se Reference Dietary Intake (RDI) for adult males and females is 55 μg per day, and the Tolerable Upper Intake level is set at 400 μg/day [59]. For all sheep products, the harvesters exceeded the Se RDI by more than a factor of seven (392.3 μg/day) and were slightly below the tolerable upper intake level of 400 μg/day. The liver protein intake alone comprised 80% of the tolerable upper limit of Se. Hypothetically, if one consumes liver protein more than once a week in the current scenario, the Se UL will be exceeded. The reported values only take into account the levels representative of sheep protein intake evaluated in this study and exclude non-subsistence and other dietary sources.

In summary, in this U mining impacted area, our calculations indicate that the Se levels found in locally harvested sheep exceeded the RDI significantly but were marginally below the established daily tolerable upper intake level. Similarly, the RDA for Mo was exceeded, while the Mo UL was not exceeded. Consuming liver once a week has exhibited exceedances in Se RDI and Mo RDA, and it is anticipated that eating more than one serving size of liver per week would cause one to exceed the Se UL. Dietary sheep intake should be adjusted to avoid exceeding the Cd PTWI and Se UL. Diversification of the overall dietary intake or minimizing the intake of high HM content foods are recommended until further research can be done. Our study was comprised of adults only; therefore, our calculations are based exclusively on adult food intake. Recommendations based on tailored research are needed for those that are more sensitive to HM exposure such as children, the elderly, pregnant women, or those with at risk health conditions. There is no dietary intake guideline for the remaining HM examined in this study.

### 4.2. Limitations

The results of this study need to be generalized with caution as the sheep sample sizes were small. Further, the sheep samples were collected from two communities that were near in distance. Still, geographical dissimilarities were apparent between the two communities. Plant heavy metal contamination levels evaluated both surface contamination and plant uptake. As this was a food chain study, we strived to examine the HM concentrations available to the primary meat staple. Comparable differences in HM levels were found in controlled and field studies utilizing unwashed plant samples [60].

## 5. Conclusions

This study highlights the importance of evaluating contamination in foods and associated food chain parts collected by harvesting communities in U mining impacted areas. Overall, public water or regulated water was shown to contain fewer heavy metals than unregulated water (private well, dam, livestock, etc.) sources for sheep. This demonstrates the importance of encouraging the use of regulated water where available. Similar to the published literature, our findings demonstrate that heavy metals have a tendency to be located in the roots rather than the above ground parts of forage. The bioaccumulation factor ratio was found to be high for Mo, Cd, and Se for most of the seven forage species evaluated, which requires further study and monitoring. Uranium concentrations in edible tissues from sheep were generally low. Our findings show that Se, Mo, and Cd may have a tendency to accumulate in sheep liver, kidney, and wool. The calculated human intake Se RDI and Mo RDA were significantly exceeded, but the upper limits for both were not exceeded. The PTWI for As, Cd, Pb and the tolerable upper limits for Se were not exceeded (marginally) in human intake. It is plausible for individuals to exceed the Se UL and Cd PTWI if sheep liver is eaten more than once a week and sheep protein 2.33 times per week, respectively. Dietary adjustments should be made according to the above recommendations. Recommendations informed by research are needed for individuals that may be more sensitive to HMs. Food sharing and selling were common with the harvesters and need further attention and characterization.

Areas for future research have been highlighted, as well as ways to refine methods for the work. Further study is needed to provide more information about the local food chain status in the community of focus. The findings from this study and future research recommendations will be shared with the communities as well as their leaders. The research findings also have the capacity to reach other mining impacted areas outside the study community. Continued research and monitoring is recommended.

## Figures and Tables

**Table 1:** Recommended standards for upper limits of potentially toxic water contaminants for sheep and maximum contaminant levels for humans.

Heavy Metal	Upper Limits ppm (μg/L)	Reference	EPA MCLs ^1^ μg/L
As	0.2 ppm (200 μg/L)	Pugh, 2002	10 μg/L ^1^
Cd	0.10–0.05 ppm (10–50 μg/L)	Pugh, 2002	5 μg/L ^1^
Pb	0.05–1 ppm (50–1000 μg/L)	Pugh, 2002; NRC ^2^, 2005	15 μg/L ^1,3^
Se	0.05 ppm (50 μg/L)	Pugh, 2002	50 μg/L ^1^
U	--	--	30 μg/L ^1,4^

^1^ Environmental Protection Agency Maximum Contaminant Levels, 2009; ^2^ National Research Council; ^3^ Pb Action Level; ^4^ Navajo Nation Environmental Protection Agency Standard, 2007.

**Table 2:** Sheep dietary requirements and/or maximum tolerable concentrations.

Heavy Metal	Requirement ^1^ or Maximum Tolerable Concentrations ^2^	Reference
As	MTC ^2^: 30 mg/kg of diet	NRC ^3^, 2005
Cd	MTC ^2^: 10 mg/kg DM ^4^ diet	NRC ^3^, 2005; 2007
Pb	MTC ^2^: 100 mg/kg diet DM ^4^	NRC ^3^, 2005; 2007
Mo	R ^1^: 0.10–0.5 ^3^ mg/kg DM ^4^	Pugh, 2002; NRC ^3^, 2007
	MTC ^2^: 5 mg/kg diet DM ^4^	NRC ^3^, 2005; 2007
Se	R ^1^: 0.10–0.20 ppm (0.10–0.20 mg/kg) diet	Pugh, 2002
	Chronic ingestion: 0.25 mg/kg BW ^5^	Pugh, 2002
	MTC ^2^: 5 mg/kg DM ^4^	NRC ^3^, 2005; 2007

^1^ Requirement; ^2^ Maximum Tolerable Concentration; ^3^ National Research Council; ^4^ Dry Matter; ^5^ Body Weight.

**Table 3:** Sheep 1 heavy metal concentrations in soil, forage, bioaccumulation factor ratio, and sheep tissue in mg/kg, dry weight.

HM	Soil (Range) *n* = 8	Shoot/Root (Range) *n* = 8/8	BF ^1^	Sheep Tissue *n* = 1
Se	0.91–1.85	0.87–1.37/1.38–1.45	0.85	3.93 Liver
				2.04/0.53 KM ^2^/KC ^3^
				1.30 Wool
Cd	0.05–0.07	0.05–0.08/0.06–0.11	1.16	0.06 Liver
				0.01 Intestine
				ng ^4^/0.01 KM ^2^/KC ^3^
As	2.33–4.53	0.84–1.41/1.40–1.54	0.34	0.66 Wool
				0.09/0.08 KM ^2^/KC ^3^
				0.08 Bone
Pb	3.91–9.07	1.15–1.94/2.03–2.72	0.26	1.12 Wool
				0.21/0.15 KM ^2^/KC ^3^
				0.20 Bone
Mo	0.10–0.13	0.79–1.16/0.30–0.37	8.53	1.20 Liver
				0.43/0.05 KM ^2^/KC ^3^
				0.08 Lung
U	0.81–0.93	0.18–0.34/0.20–0.52	0.28	0.02/ng ^4^ KM/KC
				0.06 Wool
				0.001 Liver

^1^ Bioaccumulation Factor ratio; ^2^ Kidney Medulla; ^3^ Kidney Cortex; ^4^ Negligible.

**Table 4:** Sheep 2 heavy metal concentrations in soil, forage, bioaccumulation factor ratio, and sheep tissue in mg/kg, dry weight.

HM	Soil (Range) *n* = 8	Shoot/Root (Range) *n* = 8/8	BF ^1^	Sheep Tissue *n* = 1
Se	1.17–1.88	1.13–1.61/1.81–3.50	0.80	5.93 Liver
				2.83/0.68 KM ^2^/KC ^3^
				1.88 Wool
Cd	0.06–0.17	0.08–0.16/0.20–0.29	1.53	1.02/0.01 KM ^2^/KC ^3^
				0.22 Liver
				0.04 Wool
As	1.58–3.79	0.78–1.40/1.23–1.41	0.5	0.56 Wool
				0.10/0.06 KM ^2^/KC ^3^
				0.10 Muscle
Pb	5.28–8.35	0.93–2.14/2.99–3.18	0.29	1.09 Bone
				1.07 Wool
				0.21/0.25 KM ^2^/KC ^3^
Mo	0.06–0.18	0.53–1.00/1.03–1.34	7.5	1.39 Liver
				0.66/ng ^4^ KM ^2^/KC ^3^
				0.25 Lung
U	0.48–1.15	0.08–0.31/0.28–0.42	0.23	0.001/ng ^4^ KM ^2^/KC ^3^
				0.08 Wool
				0.01 Lung

^1^ Bioaccumulation Factor ratio; ^2^ Kidney Medulla; ^3^ Kidney Cortex; ^4^ Negligible.

**Table 5:** Sheep 3 heavy metal concentrations in soil, forage, bioaccumulation factor ratio, and sheep tissue in mg/kg, dry weight.

HM	Soil (Range) *n* = 8	Shoot/Root (Range) *n* = 8/8	BF ^1^	Sheep Tissue *n* = 1
Se	2.29–2.80	1.19–2.62/2.58–3.36	0.78	3.28 Liver
				2.62/0.60 KM ^2^/KC ^3^
				3.85 Wool
Cd	0.07–0.08	0.09–0.17/0.14–0.22	1.71	0.63/0.02 KM ^2^/KC ^3^
				0.23 Liver
				0.05 Wool
As	1.20–1.75	0.55–0.63/0.82–1.06	0.42	0.71 Wool
				0.09/0.07 KM ^2^/KC ^3^
				0.14 Liver
Pb	5.23–6.37	1.36–1.58/2.42–2.62	0.25	1.90 Wool
				0.70 Bone
				0.55 Liver
Mo	0.07–1.03	1.13–1.18/0.61–1.08	8.9	1.47 Liver
				0.47/0.11 KM ^2^/KC ^3^
				0.23 Wool
U	0.36–0.38	0.09–0.10/0.14–0.23	0.51	0.002/0.001 KM ^2^/KC ^3^
				0.09 Wool
				0.01 Lung

^1^ Bioaccumulation Factor ratio; ^2^ Kidney Medulla; ^3^ Kidney Cortex.

**Table 6:** Heavy metal concentrations in soil, plants, and sheep tissue from previous studies in the study area. Heavy metal concentrations are reported for high impact areas unless otherwise specified.

Sample Type	Heavy Metal Concentration mg/kg	Reference
Soil	U: 3–8	deLemos et al., 2009 [18]
	U: 5.1 ± 2.0	deLemos et al., 2008 [41]
	U: 4.2 ± 2.0 (low impact area)	deLemos et al., 2008 [41]
	Se: 2.3/0.7 (control)	Dreesen & Cokal, 1984 [32]
Vegetation	U: 0.5–7.7	deLemos et al., 2009 [18]
Roots	U: 5.0	deLemos et al., 2009 [18]
Shoots	U: 2.4	deLemos et al., 2009 [18]
Sheep muscle	U: 0.88–0.148	Millard et al., 1986 [21]; Ruttenber et al., 1984 [22]
	Pb: 6.06–38.48	
Sheep liver	U: 1.776–2.96	Millard et al., 1986 [21]; Ruttenber et al., 1984 [22]
	Pb: 38.48–429.2	
Sheep kidney	U: 4.44–6.512	Millard et al., 1986 [21]; Ruttenber et al., 1984 [22]
	Pb: 41.44–1050.8	
Sheep bone	U: 35.52–65.12	Ruttenber et al., 1984 [22]
	Pb: 340.4	

**Table 7:** Summary of the dietary exposure of the target study population to heavy metals from sheep meat protein and organ consumption representative of one day per week.

Heavy Metal	Weekly Intake (μg/kg BW ^1^)	PTWI ^2^ (μg/kg BW ^1^)	% of PTWI	Once a Week Intake (μg)	RDI ^3^ or RDA ^4^ or UL ^5^ (μg/day)	% of RDI or RDA or UL
As	1.64	7	10.9	--	--	--
Cd	2.98	25	42.5	--	--	--
Pb	6.2	15	24.8	--	--	--
Mo	--	--	--	92.34	RDA: 45 UL: 2000	205 4.6
Se	--	--	--	392.26	RDI: 55 UL: 400	713 98

**^1^** Body Weight (reference weight 60 kg); ^2^ Provisional Tolerable Weekly Intake; ^3^ Reference Dietary Intake; ^4^ Recommended Dietary Allowance; ^5^ Tolerable Upper Intake Level.
